# Exploring Step-Heating and Lock-In Thermography NDT Using One-Sided Inspection on Low-Emissivity Composite Structures for New Rail Carbodies

**DOI:** 10.3390/s22218195

**Published:** 2022-10-26

**Authors:** Alkiviadis Tromaras, Vassilios Kappatos

**Affiliations:** Centre for Research and Technology Hellas, Hellenic Institute of Transport, 6th Km Charilaou-Thermi Road, 57001 Thessaloniki, Greece

**Keywords:** step heating, lock-in heating, thermography, infrared thermography, composite rail carbodies, CF-PET-CF sandwich, NDT, NDE, IRT

## Abstract

This paper aims to explore the qualification of step- and lock-in heating thermography as techniques capable of inspecting new composite rail carbodies following input and inspection requirements set by the rail manufacturing industry. Specifically, we studied (a) a monolithic CFRP sample (20 mm thickness) and (b) a CFRP–PET foam–CFRP sandwich (40 mm total thickness) component, that were manufactured with artificial defects, to replicate the side wall sections of a carbody. The samples proved to be very challenging to test using only one-sided inspection due to (1) exhibiting significant thickness compared to existing literature, (2) low surface emissivity and (3) that the foam core of the sandwich sample was a thermal insulating material. In addition, the sandwich sample was designed with defects on both skins. Both thermography techniques provided similar defect detection results, although step heating offered faster detection. In the case of the monolithic panel, defects up to 10 mm depth were detected, with minor detection of defects at 15 mm depth with a step-heating protocol between 90 s and 120 s overall acquisition, which was faster than the 140 s used with the lock-in technique. For the sandwich component only the front skin defects were detected, with both techniques using heating protocols between 70–120 s.

## 1. Introduction

New trends in the transportation sector show an increasing demand for energy reduction as a solution for tackling environmental issues and climate change [1]. Within this context, composite materials have found usage in the transport sector especially as structural components in aerospace [2] and automotive applications [3]. Such materials offer better mechanical properties, such as high strength and stiffness to weight ratios, over conventional materials [4]. However, in the rail sector, carbody shells have been typically manufactured using metallic components such as steel and aluminium, which leads to relative high vehicle weight. The use of composites materials in the rail sector could lead to reduction in carbody mass [4] and can potentially help the rail transport sector significantly reduce its environmental impact.

The use of carbon fibre or glass fibre components of monolithic and hybrid nature (i.e., sandwich type with foam or honeycomb core) in rail carbodies has been the subject of research by various EU-funded Shift2Rail projects such as PIVOT2 [5], GEARBODIES [6], CARBODIN [7] and Roll2Rail [8], as well as many research papers on existing prototype carbodies [4,9,10]. When substituting conventional materials with alternatives, transport system manufacturers and operators must ensure safety. Composite components may suffer failures during manufacturing as well as be subjected to harsh operational environmental conditions during their service or even maintenance [11]. Delaminations, disbonds, impact damage, core crushing, porosity or corrosion are common defects that can occur during the in-service phase of a composite component [12]. Therefore, such new composite structures would require inspection for surface and subsurface defects, using non-destructive testing (NDT) techniques either during their maintenance phase or even in-service phase.

Infrared thermography (IRT) is an NDT technique that has been well documented in applications of inspecting composite materials. IRT is capable of capturing temperature radiation of a body, which is an electromagnetic radiation. When a heat flux *Φ* falls on the surface of a body, part of it is absorbed *Φ_A_*, reflected *Φ_R_* and transmitted *Φ_Τ_* [13]. The absorbed flux increases the internal thermal energy of the body and thus its temperature.

IRT can be divided into passive and active thermography. In passive thermography the object under inspection is observed using an infrared camera without the use of any external thermal sources, and thermal variations in the specimen, which can be higher or lower than the background, are measured under its environmental temperature [11]. On the contrary, active thermography studies the object under inspection, using an external source of energy that can induce a temperature difference between defective and non-defective areas of the specimen [14]. Common types of active IRT are optical methods such as pulsed thermography, lock-in thermography (LT), electromagnetic methods, i.e., eddy current thermography, and mechanical excitation methods, i.e., lock-in vibrothermography and burst vibrothermography [14]. Ibarra-Castanedo et al. [14], Wang et al. [15], Ciampa et al. [16], Ekanayake et al. [17], D’Accardi et al. [18], Vavilov et al. [19], Liu et al. [20] and Shrestha et al. [21] describe the use of LT on composite sandwich and monolithic structures as one of the most popular IRT methods, being capable of defect detection in thicker specimens and being less prone to surface emissivity [11]. Ramos Silva et al. [22] compared the application of LT on Poly(methyl methacrylate) samples using two types of excitation. The first is an imperfect sinusoidal excitation coming from a conventional halogen heating system, while in the second case, a proportional–integral–derivative (PID) controller and light sensor have been added to the system to ensure a true sine wave excitation on the samples. This technique led to better stimulus accuracy and improved system frequency response that resulted in cleared images, especially in the case of the amplitude images.

Similarly, applications of step-heating thermography (SHT) on composite structures [23,24,25,26] are described as an alternative to pulsed thermography (PT) [19,27,28,29] for overcoming its limitations, as it is more applicable to thinner materials, and as an alternative to LT to overcome the limitations of long inspection time [23]. Apart from using heat as an excitation method in active thermography, Szymanik et al. [30] suggest the use of forced cooling as an additional excitation method that can affect heat dissipation within a material (in their case a glass fibre composite) and can cause rapid temperature change in defective areas which can be detected by an infrared camera.

IRT has found application in the inspection of large structures such as wind turbines and aircrafts [16,18,31,32,33,34,35] that may consist of thick composite components either of monolithic or hybrid nature. An innovative solution of aircraft inspection is presented by Deane et al. [32] using an unmanned aircraft vehicle (UAV) mounted with tethered halogen lamps that would be used to carry out PT. In addition, the application of IRT has become more popular over the recent years due to characteristics highlighted by [34]: (1) offering fast inspection time; (2) being a contactless inspection method; (3) offering good spatial resolution and acquisition rate; (4) affordability in terms of cost; (5) advances in thermography image processing. The aforementioned characteristics and competencies of IRT make it a suitable inspection technique for composite rail carbodies that share similar characteristics with such structures where it has already been applied to.

Table 1 presents a brief overview of IRT research that has been conducted on monolithic and sandwich composite components containing materials that could be suitable for rail carbody application, i.e., carbon fibre reinforced polymers (CFRP), glass fibre reinforced polymers (GFRP) or foam/honeycomb core materials [5,6,7,8]. Emphasis was given to thicker monolithic materials, i.e., above 5 mm.

It needs to be noted that in the identified literature regarding inspection of sandwich components listed in Table 1, all samples contained defects only in the front surface of the sample, which was the inspection side. Therefore, there was no intention of detecting defects on both sides of the sample in the aforementioned literature.

The aim of the current research is to present the application and qualification of active IRT and more specifically optical step heating and lock-in thermography techniques, where specific inspection requirements have been applied from the rail manufacturing industry for the detection of defects in new composite rail carbodies. The research presented in this paper has been carried out within the framework of the EU-funded project GEARBODIES [6]. Specifically, the paper presents inspection protocols and results using the aforementioned IRT techniques that have been applied to two thick samples of composite components manufactured with artificial defects. The two samples that were inspected are (1) a monolithic carbon fibre reinforced polymer (CFRP) component of 20 mm thickness and (2) a sandwich block consisting of CFRP skins (5 mm thickness each) and a polyethylene terephthalate (PET) foam core (30 mm thickness). These samples were designed (thickness and choice of materials) and manufactured according to input from the rail manufacturing industry to resemble structural sections of future composite carbodies, as is presented in Section 3.1.

The contribution of the current research is based on the application of IRT on the aforementioned samples that exhibit significant thickness, have thermal insulating properties (in the case of the sandwich foam core) and offer very low surface emissivity, thus making their inspection extremely challenging using only one-sided inspection. According to the authors’ knowledge and literature review, no other research has been identified covering the application of IRT on monolithic CFRP composites with 20 mm thickness or 40 mm sandwich and the combination of materials of CFRP and PET in the case of the sandwich sample. In addition, the inspection procedures that were followed were based on requirements that have been set according to input from the rail manufacturing industry. The first requirement was that the inspection of the rail carbody had to be carried out only using one-sided inspection, thus excluding the use of transmission mode thermography that can be used to overcome issues of emissivity and component thickness. Instead, the issue of emissivity was overcome by removing the heating lamps out of the IR camera’s field of view and placing them at an angle, while the camera had to be placed at larger distance from the sample. The second requirement was a time limit of 12 h that was set for inspecting both lateral sides of the carbody. Therefore, this research could be potentially useful for readers looking into identifying NDT methods for the combination of these materials in composite components as well for readers asking to what extent IRT could be used in terms of capability in detecting defects in these type of materials, as well as for new rail applications, in a more practical way.

## 2. Theory Background

The following section presents the theory behind the two IRT techniques that were applied to the inspection of the two composite samples.

### 2.1. Step-Heating Thermography (SHT)

SHT (also known as long pulse) in its conventional form is meant to be similar to (flash) pulsed thermography, with the difference of being longer in heating duration. In pulsed thermography, a short energy pulse, usually lasting milliseconds, is used to heat up an object while only the cooling phase is observed [40]. In the case of SHT, a long pulse of low-intensity heat is used, while the increase in surface temperature of the inspection area can be monitored both during and after the heating phase [41]. SHT can overcome the issue that low conductivity thick materials require high intensity or long duration pulses [42].

### 2.2. Lock-In Thermography (LT)

LT is another method that was applied in the inspection of the composite samples. It is a popular legacy method that is based on periodic deposition of heat on the object under inspection. The term lock-in refers to the need to monitor the exact time dependency between the output and the reference input signal [41] caused by the presence of defects. Typically, sinusoidal waves are used in LT, thus giving the advantage that response shape and frequency are preserved [14]. According to Maldague [41], the depth range that LT can detect defects is proportional to the modulation frequency.

This relationship is provided by Equation (1) [14]:

(1) $$z=C_{1}*\mu =C_{1}\sqrt{\frac{a}{\pi \;f}}$$

*z* is the probing depth (m), *C*_1_ is an empirical constant, *μ* (m) is the thermal diffusion length that determines the rate of decay for the thermal wave as it penetrates a material, α is the thermal diffusivity (m^2^/s) (*α* = *k*/*ρC_ρ_*, where *k* (W/mK) is thermal conductivity of the material, *ρ* is the density (kg/m^3^) and *C_p_* (J/kgK) is the specific heat at a constant pressure), and *f* (Hz) is the frequency [14]. The value of *C*_1_ varies and depends on the type of data the user wants to inspect. For inspecting amplitude data, *C*_1_ is estimated to be 1 in value or 1.82 for phase data [14]. The diffusivity of CFRP is 4.3 × 10^−7^ m^2^·s^−1^ [43]. Equation (2) can help determine the period of the sinusoidal thermal wave that the lock-in technique should use, which is 1/*f*.

(2) $$f=\frac{C_{1}{}^{2}*\;a}{\pi Z^{2}}$$

## 3. Experimental Setup and Procedures

### 3.1. Samples

#### 3.1.1. Sample Properties

**Figure 1:**
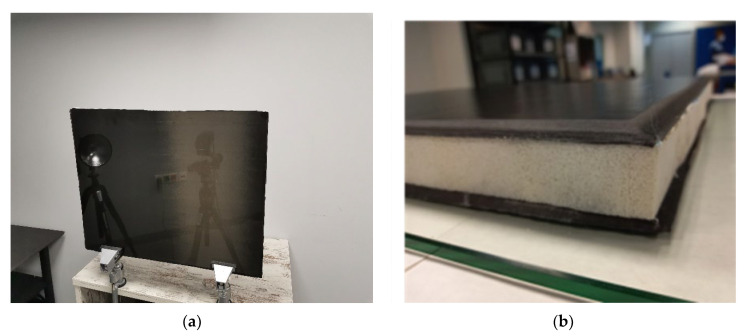
Photos of the CF-PET-CF sandwich sample: (**a**) very smooth low emissivity inspection side and (**b**) side view presenting the foam core and CF skins.

Two composite samples were manufactured according to input from the rail industry. Both samples are meant to replicate side wall sections of a new prototype composite rail carbody. Specifically, the monolithic carbon fibre (CF) component will find use in areas around the windows sections, while the CF-PET-CF sandwich components will be used for all the remaining lateral side wall sections. General information regarding the samples is presented on Table 2, while Figure 1 presents a photo of the CF-PET-CF sandwich sample. The thickness of the samples was one of the main challenges, while in the case of the sandwich, the PET foam core, apart from providing rigidity to the structure, is used for the thermal and noise insulating properties that it can offer to the rail carbody. Thus, detecting defects on both skins of the sandwich sample using one-sided inspection was extremely challenging.

The two samples were manufactured using pre-impregnated plies of CFRP, stacked on top of each other, to create the desired thickness of the monolithic block and respectively the CF skins of the sandwich. The surface smoothness of the CF surface of the samples, as seen on Figure 1, was created as such, on purpose, in order to replicate the surface finish that a composite rail carbody will have (prior to any paint or decals being applied to its surface), according to input from the rail industry.

#### 3.1.2. Sample Defects

Both samples were manufactured with artificial inserts made of Teflon^®^ tape in order to simulate defects such as delaminations and disbonds that can appear on the side wall sections of a train. Specifically, such defects can be caused by impact damage from rail ballasts or other flying objects as the train travels through the rail tracks at high speed [10]. In the case of the sandwich component, impact damage, apart from delaminations, can cause disbond between the foam core and the CF skin. Alternatively, delaminations can occur at various monolithic structural sections of the carbody, including the areas around the windows, due to acceleration and breaking of the train.

**Figure 2:**
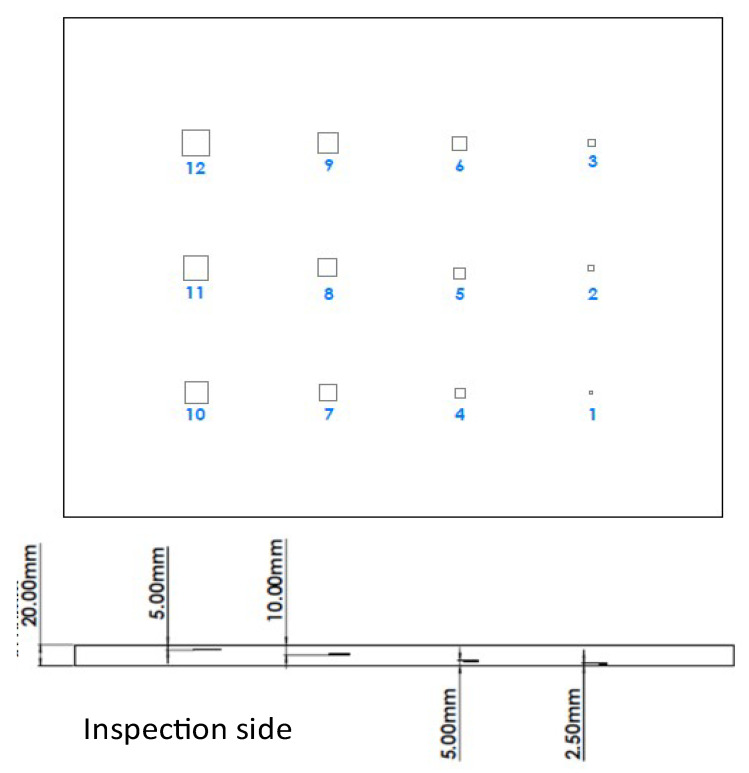
Schematic of the monolithic plate sample.

**Figure 3:**
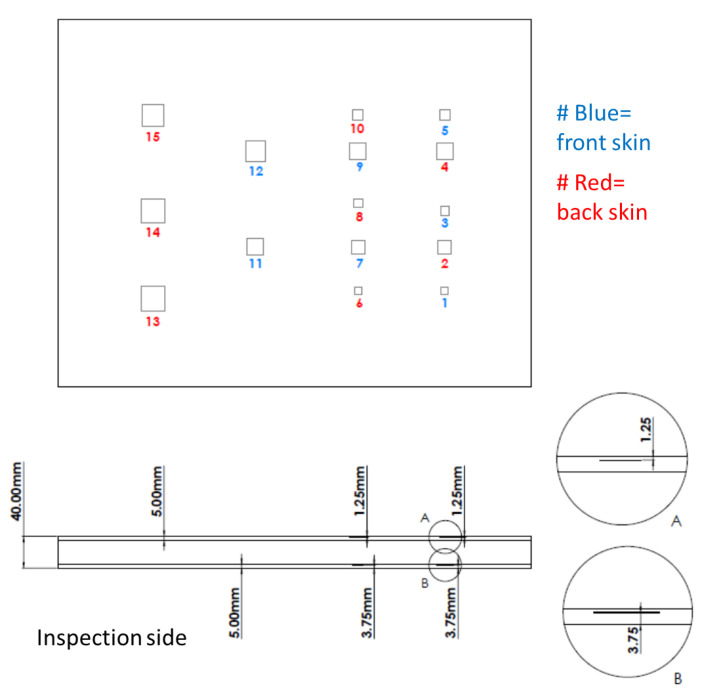
Schematic of sandwich sample.

In order to test the ability of IRT to detect such defects, the artificial Teflon^®^ inserts of different dimensions were introduced at various depth levels of the samples. Teflon^®^ inserts were placed between the foam core and the skins to replicate such disbonds by creating a lack of adhesion between the two materials. Figure 2 and Table 3 present the depth of the artificial defects on the monolithic plate ranging from 2.5 mm up to 15 mm and their dimensions, respectively. Figure 3 and Table 4 present the equivalent data regarding the Teflon^®^ inserts’ dimensions and their location in the sandwich sample. The thickness of the Teflon^®^ inserts for the monolithic CF sample was 1 mm and 0.22–0.24 mm for the CF-PET-CF sandwich sample, respectively. For the sandwich sample the front skin is the inspection side. The range of dimensions of the inserts and the depth at which they were introduced were verified by the rail manufacturing industry.

### 3.2. Thermographic Equipment

**Figure 4:**
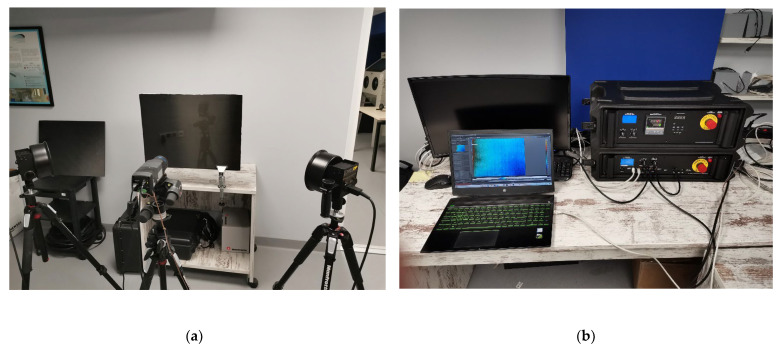
IRT inspection equipment. (**a**) Halogen lamps and IR camera, (**b**) PC and IRT controllers.

The IRT equipment that was used (Figure 4) consists of a set of two halogen lamps, 1 kW each, an IRT controller manufactured by Visiooimage Inc., Québec, Canada [44] and an FLIR A655sc uncooled microbolometer with a 45° lens. The IRT controller offers a variety of functions, acting as a hub that allows connectivity between IR camera, halogen power and duration, and PC for image acquisition and storage, remotely controlled by the Telnet protocol.

FLIR’s ResearchIR software was used for image acquisition, while all the image processing was carried out on IR View software supplied by Visiooimage Inc., Québec, Canada [44].

### 3.3. Procedure

The following section presents the thermography protocols and procedures that were applied for the inspection of the two composite samples. Reflection mode IRT was used based on the requirements set by the rail industry for one-sided inspection of the carbody. In addition, the approach that was followed in the experimentation was to try and detect all defects at once within the sample using a single heating protocol and acquisition that would cover the sample’s entire surface area, due to the 12 h time limit imposed for the overall carbody inspection.

#### 3.3.1. Issues Encountered

**Figure 5:**
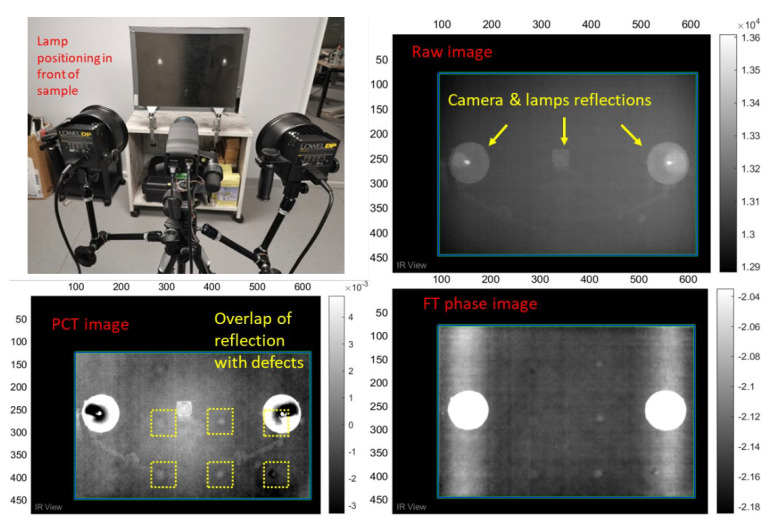
Reflections due to low emissivity of the samples and their effect on acquisitions.

**Figure 6:**
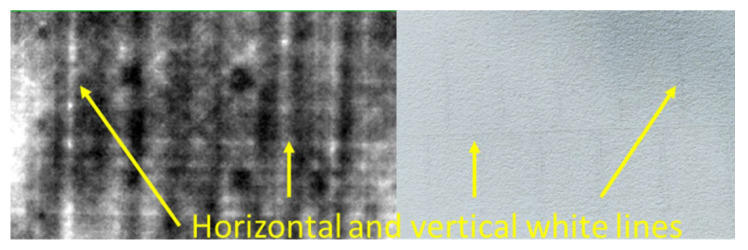
IR image of the canals located in the PET foam core.

The inspection of the two samples proved to be challenging due to their large thickness and properties. The issues that were encountered are explained below. Low emissivity of the samples: The smoothness of the inspection CFRP surfaces created an almost mirror-like surface finish, causing IR reflections from objects (i.e., IR camera, halogen lamps, metal tripods, electric appliances, people) in the vicinity of the sample being detected by the IR camera. When the halogen lamps were initially placed directly in front of the samples, their heat signature and reflection would be part of the acquisition, thus covering defects and creating contrast issues in the raw and processed images, as seen in Figure 5. Thus, the heating lamps had to be moved out of the camera’s field of view and be placed at a 45° angle, while the camera was also moved further away from the inspection surface at 90 cm away from the sample’s surface (see Table 5). All objects that could also cause reflections in the room where the experiments took place had to be moved away as well. PET foam sandwich core: The PET foam sandwich core has vertical and horizontal canals running throughout its structure, which are used for helping the resin flow better during the manufacturing process of the component. These canals were actually visible in the IR images, as seen in Figure 6.

#### 3.3.2. Optical Lock-In Acquisition Protocols

Table 6 presents the heating protocols that were used for the lock-in acquisitions on the monolithic sample. The Visiooimage IRT controller uses power rates to control the amount of power and consequently heat projected by the heating lamps. The min and max power rates indicate the amount of power used to heat the sample. A rate of 255/255 is equivalent to ~2000 W at 240 V or ~1.800+ W at 230 V, which was the case during these acquisitions. The min and max rates are also provided to present the values that were used to create the sinusoidal thermal wave. The ‘heating duration’ indicates the duration that the sample was subjected to heat, while the ‘period duration’ is the period of the sinusoidal thermal wave. Each LT protocol was meant to probe to a different depth based on Equation (2). Acquisition ‘LM1′ (lock-in monolithic) is estimated to be able to probe up to 4 mm depth, LM2 up to 5 mm and LM3 up to 15 mm, respectively.

Table 7 presents the optical lock-in heating protocols that were used in the inspection of the sandwich sample. However, most of the acquisitions had noise due to the resin canals of the PET foam creating their own thermal contrasts and interfering with the CF skin, as previously shown in Figure 6. The majority of the protocols probed for defects up to 5 mm depth, within the whole front skin thickness, with the aim of improving the thermal contrasts of the artificial defects on the front skin. This was due to the fact that the inspections identified defects only on the front sandwich skin, as is presented in the Results section. The only protocol that probed for a full thickness inspection is LS6 (lock-in sandwich), which is estimated to take 1.45 h.

#### 3.3.3. Step-Heating Acquisition Protocols

Step-heating was the second technique that was applied for inspecting the two composite samples. During the experiments carried out in this research, both cooling and heating phase were observed in the acquisitions. This was due to the fact that observing only the cooling phase of the acquisition (as performed in pulsed thermography acquisition procedures) did not provide any significant results.

The heating protocols from the application of SHT on the monolithic sample are presented in Table 8. In most cases an equal observation time to that of heating was used, although in some cases (SHM4 and SHM5) (step-heating monolithic) a smaller observation time was used to compensate for longer heating duration. The initial aim was to experimentally identify the heating duration of the protocol in order to start obtaining better detection results, while the second goal was to narrow down the heating duration and observation time. Some further acquisitions were performed to identify how many defects can be detected by using lower overall acquisition and heating duration.

Table 9 presents the heating protocols that were applied to inspect the sandwich sample using SHT. In an effort to reduce reflections and improve the signal-to-noise ratio (SNR) of the thermal images, the sandwich sample was painted using a black matte water-based paint. The idea was to move the halogen lamps orthogonally to the sample and observe any changes in the SNR of the images. Direct placement of the lamps in front of the sample, even with the paint, caused hot spots. Although the latter were significantly smaller compared to Figure 5, they were observed in the IR images and overlapped with defects. Although no significant SNR improvements were made due to the paint, the reflections were reduced, and the halogen lamps could be moved closer to the sample and at a better angle. However, painting the sample was only considered as a lab solution and not viable for future usage on a full-scale rail carbody at a maintenance facility. Thus, better placement of the lamps was considered instead.

#### 3.3.4. Image Processing Techniques

All acquisitions were pre-processed, using the IR View software, by cutting out the edges of the image that were not required in order to focus on the defective areas of the samples as well as to remove areas with higher temperatures that could affect the post-processing techniques and the image contrasts. Cold image subtraction was also performed to remove the reflection of the camera appearing in the first images of the acquisition. However, in some cases this process reduced the brightness of the post-processed images, and since it was not overlapping with any of the defects it was decided not to carry out this technique. Post processing of the acquisitions was carried out using three main techniques offered by IR View software: principal component thermography (PCT) and amplitude (FTA) and phase (FTP) signal of the Fourier Transform. In addition, a median and gaussian filter was applied to all thermograms to remove salt and pepper noise. No further theory or algorithms were specifically developed in this paper; only well-known methods of defect detection by IRT were used.

The PCT technique was proposed by Rajic [45] and uses singular value decomposition (SVD) to reduce thermographic data, which can be considered as a 3D matrix consisting of N thermograms in time to a set of orthogonal functions that provide spatial and temporal information [16]. The other two techniques used for post processing the thermograms are FTA and FTP, which were performed in the time dimension of the IR sequence of images in the IR View software. These two techniques analyse a signal into its constituent frequencies in terms of amplitude and phase differences, respectively. The thermography acquisition data, consisting of a series of thermograms in time, are inserted into a 3D array with x,y dimensions where a fixed pixel location is analysed across all the thermograms of the acquisition. The z-dimension of the 3D array consists of the time that the thermograms were recorded during the acquisition. The FFT technique is used to produce, per pixel, an overtime function that will provide the constituent frequencies of the change in the pixel’s absolute temperature value across the acquisition. Consequently, the result of these functions is an amplitude and phase delay map per frequency.

## 4. IRT Inspection Results

The following section presents the results from the application of LT and SHT on the two composite samples. The results cover the number of defects that were detected in the raw image as well as using image processing techniques. In the majority of the acquisitions the raw, i.e., unprocessed images provided minor or no detection of the Teflon^®^ defects.

### 4.1. Optical Lock-In Results from Monolithic CFRP Sample

**Figure 7:**
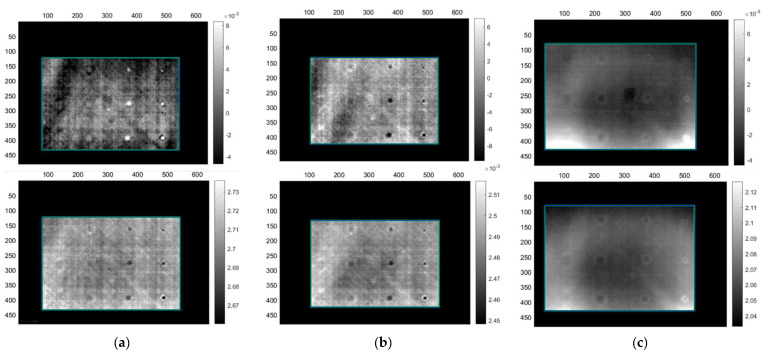
Lock-in thermography PCT (**top**) and FT phase (**bottom**) images from inspection of the monolithic CFRP sample. (**a**) Images from acquisitions LM1, (**b**) LM2 and (**c**) LM3.

The monolithic sample has proven to be relatively easier to inspect compared to the sandwich, although its 20 mm thickness was the main challenge. Although, the Teflon^®^ inserts were easy to detect due to being 1 mm thick, their thermal properties seemed to create detection issues during long-duration heating protocols. A possible explanation is that the Teflon^®^ tape that was used inhibited similar thermal properties with the CF material. However, this could not be verified due to such data being unavailable from the Teflon^®^ tape manufacturer. Table 10 presents the number of defects that were detected using the different image processing methods and the maximum depth that the heating protocol allows the user to observe defects. Acquisitions LM1 and LM2 (Figure 7a,b respectively) show that detection of defects up to 5 mm in depth is possible using an overall heating time of up to 120 s. Defects appear as black or white dots (depending on the type of image), while shallower defects are located on the right side of the image and become progressively deeper by each column of defects that appears. Although LM2′s heating period is used to probe for a depth of 5 mm, defects up to 10 mm begin to become visible. For the detection of defects from 10 up to 15 mm depth, a much longer heating wave period is required, hence LM3 was performed. However, long-duration heating seems to affect the overall detection of all the defects and cause the Teflon^®^ inserts to behave in the same manner as the CF material. The white outlines that can be observed around the defects are blisters, caused by the Teflon^®^ inserts stretching the CF plies during the manufacturing process, while diagonal or horizontal lines are caused by the direction of the CF fibres of the material. As observed in Figure 7c, the two deeper columns of defects on the left side of the images begin to start being noticeable, although the last column at 15 mm only gives a very low signal-to-noise ratio (SNR).

### 4.2. Optical Lock-In Results from CF-PET-CF Sandwich Sample

**Figure 8:**
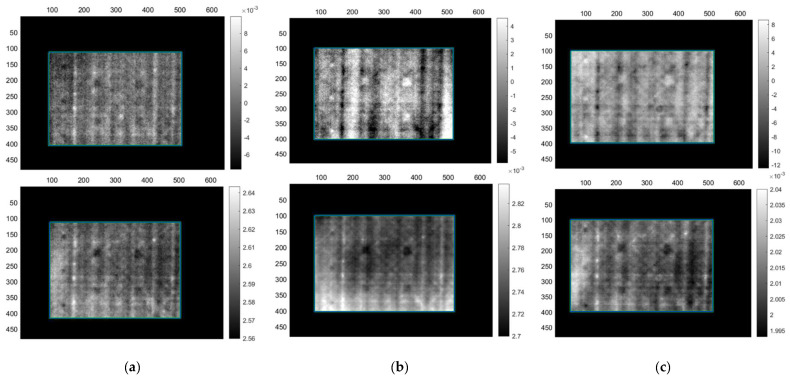
Lock-in thermography PCT (**top**) and FT phase (**bottom**) mirror images from inspection of the CF-PET-CF sandwich sample. (**a**) Images from acquisitions LS1, (**b**) LS2 and (**c**) LS5.

The sandwich sample was the most challenging of the two samples. Table 11 presents the lock-in results for this component. The main obstacle in this sample was the PET foam itself, which is a thermal insulator. Thus, only the defects located on the front skin, where the IR equipment was located, were able to be detected. No heating protocol was able to detect any defects at the back skin of the sandwich component. The majority of the acquisitions, as presented in Figure 8, varied in terms of lock-in heating wave period, ranging between 65–70 s and using either two or three wave periods in total in order to improve the contrast of the artificial defects. The specific heating wave periods are enough for probing just beyond 5 mm depth. Consequently, the total heating time duration varied between 140 s to 260 s. In order to probe beyond the front skin and the foam core, a heating wave period of 2620 s is required, making a two-period heating duration equal to 5240 s. Such inspection times for an area of around 600 mm × 460 mm, as big as the sample, would make impossible a 12 h inspection time for the two side walls of a rail carbody shell. Regardless of the inspection duration time an additional acquisition LS6 was performed.

LS6 provided much worse results compared to the other acquisitions, with no defects being detected at all, even in the front skin, possibly attributed to the amount of heat and the Teflon^®^ defects overheating during the 1.45 h heating protocol. Further experimentation with long heating protocols could be part of future work, however, the extremely long inspection time makes them impractical.

### 4.3. Step-Heating Results from the Monolithic CFRP Sample

**Figure 9:**
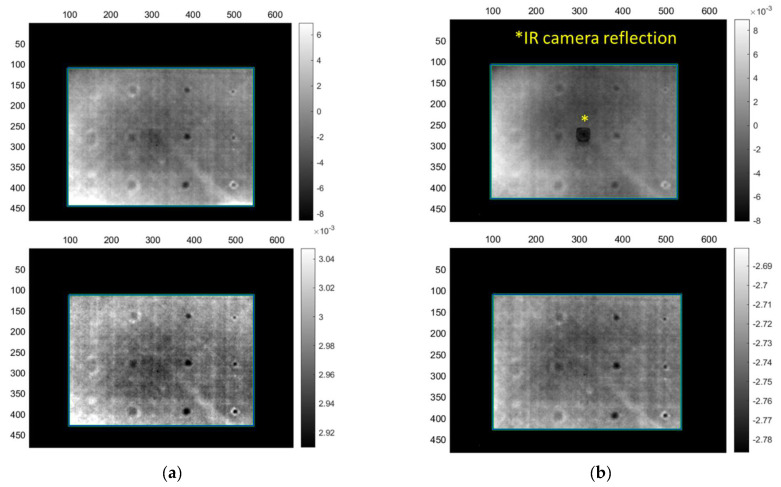
Step-heating thermography PCT (**top**) and FT phase (**bottom**) images from inspection of the CFRP monolithic sample. (**a**) Images from acquisition SHM5, (**b**) SHM6.

Table 12 presents the results from the application of SHT on the monolithic CFRP sample. Overall, the results are similar to that of the lock-in thermography, with small differences in the amount of noise in the images. Although step heating is not bound by a direct relationship between probing depth and heating time like lock-in thermography, the overall inspection times are very similar due to the fact that in this case both heating and cooling phases were observed. Acquisitions SHM5 and SHM6, with the highest number of detected defects, are presented in Figure 9. These two acquisitions range between 50–90 s heating time with an overall acquisition time of 100–120 s. These acquisitions have proven that they can detect defects up to 10 mm depth, while also providing low SNR detection of deeper defects at 15 mm.

### 4.4. Step-Heating Results from the CF-PET-CF Sandwich Sample

**Figure 10:**
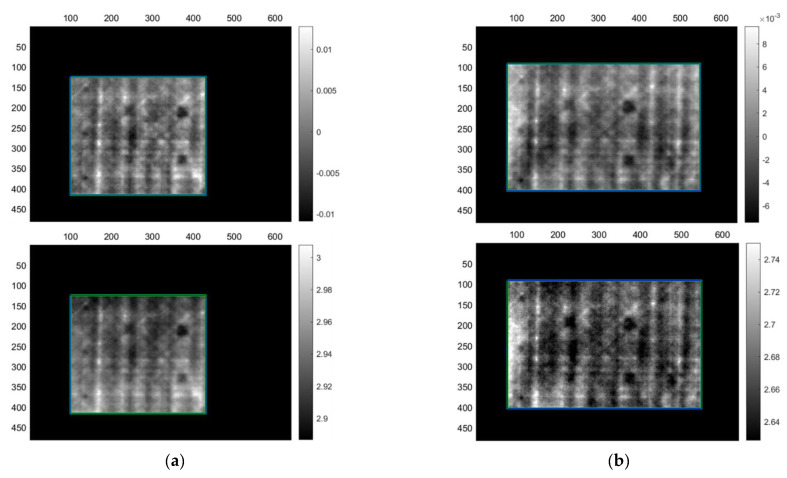
Step-heating thermography PCT (**top**) and FT phase (**bottom**) mirror images from CF-PET-CF sandwich sample. (**a**) Images from acquisition SHS3 without paint and (**b**) SHS5 with paint.

Step-heating results from the inspection of the sandwich sample are presented in Table 13. Step heating seems to offer better results compared to the images provided by lock-in for this sample. Regardless, step heating also provided the same results with lock-in in terms of the number of defects that were detected. Once again, only the front skin defects were detected, without any indication of detection of the back skin defects even with very low SNR. Furthermore, the foam resin canals are visible with this technique as well, causing the creation of their own contrasts. Figure 10 presents acquisitions SHS3 and SHS5. SHS3 is a simple acquisition using 85 s of step heating and an equal observation time during the cooling phase, while SHS5 is after the sample has been painted using a heating protocol of 70 s heating. The addition of the paint made no significant improvement in defect detection or SNR. Heating protocols below 60 s did not offer adequate results.

## 5. Conclusions

The current paper presented the application of SHT and LT IRT on monolithic and sandwich composite components that will find structural usage on future composite rail carbodies, with the aim of presenting to the reader the capabilities of IRT as an inspection method for such specific application (although not limited). The inspection procedures had to comply with specific requirements set by the rail manufacturing industry that required only one-sided inspection as well as time limitations. The paper presented the challenges, signal processing results and limitations from the application of these IRT techniques. A gap in literature was identified regarding the use of IRT for inspecting the aforementioned components with low surface emissivity, such thickness or the combination of the sandwich materials.

The key challenges for the IRT inspections were the reflections caused by the low-emissivity shiny surface of the CFRP, the thickness of the samples and the thermal insulating material used for the sandwich core. Being restricted to reflection mode IRT, meant that the only solution to overcome this issue was to place the halogen lamps out of the IR camera’s field of view and at an angle. Hence, the use of longer heating protocols was another solution for the thickness of the samples. Furthermore, the Teflon^®^ inserts also proved to be problematic due to inhibiting similar thermal behaviour with the CFRP material. In addition, the thermal properties of the PET foam meant that the core acted almost as the end of the component, thus making defects visible only in the front skin.

SHT and LT provided similar results, although step heating offered better results in an overall shorter duration. For the monolithic sample, both techniques detected defects up to 10 mm depth and gave indication of the defects at 15 mm depth. Overall, the inspection of the monolithic CFRP sample was easier due to the thickness of the 1 mm Teflon^®^ inserts as well as that they consisted of a single material. For the sandwich component, only defects on the front skin were detected using both IRT techniques, limiting the detection to 5 mm depth. However, the SNR of the defects was low, possibly due to the thin Teflon^®^ inserts. In retrospect, defects of larger size and thickness could potentially make detection easier and be tested in future work.

The results from this research conclude that SHT and LT are viable NDT techniques that can be used to inspect composite rail carbodies. However, further experimentation is required to improve detection using either other materials for creating artificial defects or inducing actual defects (i.e., impact damage or drilled holes) on the samples. Further assessment of the capabilities of these IRT techniques can be conducted using larger defects to establish a relationship between defect size and detection. Furthermore, part of future work is also to explore transmission mode IRT, simply to verify any potential changes in detecting all the defects throughout the thickness of the components.

Scalability of the inspection procedures to accommodate the scale of a commercial rail carbody or other transport vehicles that could use such composite components is needed. Using an IR camera with enough resolution and the same heat sources, larger carbody areas could be inspected in the same amount of time in order to decrease overall inspection time. Automation of the IRT techniques and their integration into an automated inspection platform is part of the ongoing research within the GEARBODIES EU project.

## Figures and Tables

**Table 1:** Brief overview of research covering IRT inspection of thick monolithic and sandwich composite components listed by size of thickness.

Type	Thickness (mm)	Reference
	Monolithic	
GFRP	11–34 step wedge	[26]
	12.7	[23]
	6 and 10	[30]
	8	[36]
CFRP	15	[37]
	10	[20]
	7.3	[25]
	**Sandwich**	
Perforated CFRP-epoxy layer-aluminium honeycomb core-epoxy layer-nonperforated CFRP-thermal insulation layer covered with stainless steel thin sheet **	CFRP: 0.8 each, core: 7, thermal insulation layer covered with thin stainless sheet: 7, epoxy adhesive: 0.2	[31]
CFRP-Aramid honeycomb core-GFRP	CFRP: 2, core: 10, GFRP: 2	[21]
GFRP-aluminium honeycomb core-GFRP	Skin: 0.5, core: 10	[38]
CFRP-aluminium honeycomb core-CFRP	Skin: N/A, core: 7.6	[14]
Kevlar-PVC foam core-Kevlar	Skin: N/A *, core: 5	[39]

* N/A = not available. The authors did not specify thickness. ** Describes the different layers of an inner fixed structure bond panel of a jet engine nacelle.

**Table 2:** Properties of the composite samples.

Properties	Monolithic	Sandwich
Composition	CFRP	CFRP skins + PET foam core
Overall component thickness	20 mm	40 mm
CF layer direction	Unidirectional 0/90, ±45	Unidirectional 0/90, ±45 ^1^
CF skin thickness	-	5 mm (front & back)
PET core thickness	-	30 mm
Dimensions	480 × 600 mm	460 × 600 mm

^1^ For both front and back CF skin.

**Table 3:** Dimensions of the artificial Teflon defects for the monolithic sample.

Ref ID	1	2	3	4	5	6
Size (mm)	3 × 3	5 × 5	7 × 7	9 × 9	11 × 11	13 × 13
**Ref ID**	**7**	**8**	**9**	**10**	**11**	**12**
Size (mm)	15 × 15	17 × 17	19 × 19	21 × 21	23 × 23	25 × 25

**Table 4:** Dimensions of the artificial Teflon defects for the sandwich sample.

Ref ID	1	2	3	4	5	6	7	8
Size (mm)	9 × 9	17 × 17	11 × 11	21 × 21	13 × 13	9 × 9	17 × 17	11 × 11
Skin	Front	Back	Front	Back	Front	Back	Front	Back
**Ref ID**	**9**	**10**	11 *	12 *	13 *	14 *	15 *	
Size (mm)	21 × 21	13 × 13	21 × 21	25 × 25	27 × 27	29 × 29	31 × 31	
Skin	Front	Back	Front	Front	Back	Back	Back	

* Indicates disbond defects.

**Table 5:** Distances between equipment.

	Distance (cm)
Camera to sample surface	90
Lamps to sample surface	105

**Table 6:** Optical lock-in acquisition protocols applied to the monolithic CFRP sample.

Acquisition No.	Min Power Rate	Max Power Rate	Heating Duration (s)	Period Duration (s)
LM1 ^1^	20/255	255/255	80	40
LM2	20/255	200/255	120	60
LM3	50/255	200/255	1100	550

^1^ LM = lock-in monolithic.

**Table 7:** Optical lock-in acquisition protocols applied to the CF-PET-CF sandwich sample.

Acquisition No.	Min Power Rate	Max Power Rate	Heating Duration (s)	Period Duration (s)
LS1 ^1^	20/255	200/255	120	30
LS2	50/255	150/255	1200	600
LS3	20/255	255/255	210	70
LS4	20/255	255/255	260	65
LS5	20/255	255/255	140	70
LS6	20/255	170/255	5240	2620

^1^ LS = lock-in sandwich.

**Table 8:** Step-heating acquisition protocols applied to the monolithic CFRP sample.

Acquisition No.	Power Rate	Heating Duration (s)	Acquisition Duration (s)
SHM1 ^1^	255/255	75	130
SHM2	255/255	80	160
SHM3	255/255	65	130
SHM4	255/255	85	120
SHM5	255/255	90	120
SHM6	255/255	50	100
SHM7	255/255	30	60

^1^ SHM = step-heating monolithic.

**Table 9:** Step-heating acquisition protocols applied to the sandwich CF-PET-CF sample.

Acquisition No.	Power Rate	Heating Duration (s)	Acquisition Duration (s)
SHS1 ^1^	250/255	70	140
SHS2	250/255	75	150
SHS3	250/255	85	170
SHS4 paint ^2^	255/255	70	140
SHS5 paint	200/255	70	140
SHS6 paint	255/255	50	100
SHS7 paint	255/255	60	120
SHS8 paint	250/255	40	80

^1^ SHS = step-heating sandwich. ^2^ Paint = the sample has been painted with black matte water-based paint.

**Table 10:** Optical lock-in results from the inspection of the monolithic CFRP sample.

Acquisition No.	Raw	PCT	FT Amp	FT Phase	Detection Depth Limit (mm)
LM1 ^1^	0/12	6/12	6/12	6/12	5
LM2	0/12	9/12	9/12	9/12	5
LM3	0/12	9/12	0/12	9/12	10–15

^1^ LM = lock-in monolithic.

**Table 11:** Optical lock-in results from the inspection of the CF-PET-CF sandwich sample.

Acquisition No.	Raw	PCT	FT Amp	FT Phase	Detection Depth Limit (mm)
LS1 ^1^	0/15	7/15	4/17	7/15	5
LS2	0/15	7/15	2/17	7/12	5
LS3	0/15	7/15	2/15	7/15	5
LS4	0/15	7/15	2/15	7/15	5
LS5	0/15	7/15	2/15	7/15	5
LS6	0/15	0/15	0/15	0/15	0

^1^ LS = lock-in sandwich.

**Table 12:** Step-heating results from the inspection of the monolithic CFRP sample.

Acquisition No.	Raw	PCT	FT Amp	FT Phase	Detection Depth Limit (mm)
SHM1 ^1^	6 */12	9/12	6 */12	6/12	10
SHM2	0/12	9/12	6 */12	9/12	10
SHM3	0/12	9/12	9 */12	9/12	10
SHM4	0/12	9/12	6/12	9/12	10
SHM5	9 */12	9/12	6/12	9/12	10
SHM6	6 */12	9/12	6/12	9/12	10
SHM7	0/12	6/12	6/12	6/12	5

^1^ SHM = step-heating monolithic; * indicates low SNR.

**Table 13:** Step-heating results from the inspection of the CF-PET-CF sandwich sample.

Acquisition No.	Raw	PCT	FT Amp	FT Phase	Detection Depth Limit (mm)
SHS1 ^1^	0/15	4/15	0/12	7 */15	5
SHS2	0/15	7/15	0/15	7/15	5
SHS3	0/15	7/15	0/15	7 */15	5
SHS4 paint ^2^	0/15	4/15	0/15	7 */15	5
SHS5 paint	0/15	7/15	0/15	7/15	5
SHS6 paint	0/15	4 */15	0/15	4 */15	-
SHS7 paint	0/15	4 */15	0/15	4 */15	-
SHS8 paint	0/15	4 */15	0/15	4 */15	-

^1^ SHS = Step-heating sandwich. ^2^ Paint = the sample has been painted with black matte water-based paint; * indicates low SNR.

## Data Availability

Data sharing not available.
